# Fungal infections in burn patients: The rise of *Fusarium* as the most prevalent in a burn center in Mexico City

**DOI:** 10.1093/mmy/myaf059

**Published:** 2025-07-21

**Authors:** Maura Cecilia González Guerrero, Jaime Arturo Mondragón Eguiluz, María de Lourdes García Hernández, Guillermo Cerón González, Claudia Adriana Colín Castro, Esteban Cruz Arenas, María de Lourdes Guerrero Almeida, Edgar Samuel Vanegas Rodríguez, Rafael Franco Cendejas, Luis Esaú López Jácome

**Affiliations:** División de Infectología, Instituto Nacional de Cancerología, CP. 14080, Mexico City, Mexico; División de Infectología, Instituto Nacional de Rehabilitación Luis Guillermo Ibarra Ibarra, CP. 14389, Mexico City, Mexico; Laboratorio de Microbiología Clínica, División de Infectología, Instituto Nacional de Rehabilitación Luis Guillermo Ibarra Ibarra, CP. 14389, Mexico City, Mexico; Laboratorio de Microbiología Clínica, División de Infectología, Instituto Nacional de Rehabilitación Luis Guillermo Ibarra Ibarra, CP. 14389, Mexico City, Mexico; Laboratorio de Microbiología Clínica, División de Infectología, Instituto Nacional de Rehabilitación Luis Guillermo Ibarra Ibarra, CP. 14389, Mexico City, Mexico; Unidad de Vigilancia Epidemiología, División de Infectología, Instituto Nacional de Rehabilitación Luis Guillermo Ibarra Ibarra, CP. 14389, Mexico City, Mexico; Gerencia médica, Epic-Cro Research, CP. 04980, Mexico City, Mexico; División de Infectología, Instituto Nacional de Rehabilitación Luis Guillermo Ibarra Ibarra, CP. 14389, Mexico City, Mexico; Subdirección de Investigación Biomédica, Instituto Nacional de Rehabilitación Luis Guillermo Ibarra Ibarra, CP. 14389, Mexico City, Mexico; Departamento de Biología, Facultad de Química, Universidad Nacional Autónoma de México, CP. 04510, Mexico City, Mexico; Laboratorio de Microbiología Clínica, División de Infectología, Instituto Nacional de Rehabilitación Luis Guillermo Ibarra Ibarra, CP. 14389, Mexico City, Mexico

**Keywords:** Burn patients, molds, invasive fungal infection, *Fusarium* spp, *Aspergillus* spp

## Abstract

Burns are among the most devastating traumatic injuries. The primary risk of infection stems from disruption of the primary barrier, the skin. For patients with deep burns, loss of the dermis is the main risk factor for systemic bacterial and fungal infections, which occur in 70% and 20%–25% of cases, respectively. Meanwhile, viral infections occur in 5%–10% of cases. Fungal infections are associated with mortality rates ranging from 33% to 60%. This study is a retrospective, cross-sectional analysis conducted at the Centro Nacional de Investigación y Atención de Quemados at the Instituto Nacional de Rehabilitación Luis Guillermo Ibarra Ibarra in Mexico City. The study examined all fungi recovered from biopsies of burn patients from July 2011 to July 2023. A total of 63 cases were included, predominantly flame burns (77.78%), with *Fusarium* spp. (53.97%) as the predominant fungal genus associated with infection, followed by *Aspergillus* spp. (19.04%). Most patients had third-degree burns, and the mean total body surface area burned was 46.2%. This study aims to describe the epidemiology and distribution of mold infections in a tertiary care center for burn patients in Mexico City from July 2011 to July 2023.

## Introduction

Burns are among the most devastating traumatic injuries, affecting the skin and underlying tissues. The etiology of burns can be categorized into five distinct classifications: heat, direct flame, radiation, electricity, or chemical agents. Annually, approximately 180 000 mortalities worldwide are attributable to burns.^1,2^ In Mexico, the Dirección General de Epidemiología has reported that in 2023, there were 66 472 cases of burns.^3^ The primary risk of infection is the absence of the primary barrier, which is the skin. The loss of the dermis in patients with deep burns is the primary risk factor for systemic bacterial and fungal infections, occurring in 70% and 20%–25% of hospitalized cases, respectively, while viral infections occur in 5%–10% of cases.^4^ In the immediate aftermath of a burn injury, the wound site is characterized by a sterile environment. However, the lesion is rapidly colonized mainly by *Staphylococcus* spp. (formerly known as coagulase-negative Staphylococci); after 48 hours, Gram-negative bacilli establish infection after the second week of the injury, and after the third week, invasive fungal infection can be found mainly by yeasts.^4,5^ Inhalation injury refers to damage to the respiratory tract induced by direct thermal injury and chemical irritation, which leads to primary pneumonia and ventilator-associated pneumonia. This condition increases the probability of death from 40% to 60%.^6^ In addition, inhalation injury is related to fungal infections, along with renal dysfunction, use of broad-spectrum antibiotics, and gastrointestinal complications.^7,8^ Among invasive fungal infections (IFI), the most frequently reported fungi are yeast and *Aspergillus* spp., followed by Mucorales, *Fusarium* spp., and *Scedosporium* spp.^9^ The highest mortality rates were observed in cases involving both yeast and *Aspergillus* spp. (33%);^10^ although this figure may be an underestimation. This is due to the paucity of epidemiological data concerning fungal infections in burn patients, with infections associated with molds reported in approximately 1.7% of cases.^11^ Early excision and wound closure are the prevailing standard of care, as evidenced by the literature that demonstrates a reduction in complications, such as infections, in severe cases.^12^ This risk is mitigated when debridement and excision are performed within the initial three days post-injury.^13^

A paucity of literature exists on the distribution of mold species implicated in IFI in patients with deep burns worldwide, including Mexico. This is particularly challenging due to the protracted nature of the procedures involved, ranging from inoculation to identification. Culture remains the prevailing standard, exhibiting a sensitivity range of 50%–70%.^10^ It is noteworthy that a limited number of clinical microbiology laboratories have adopted the mycology section. This is particularly evident within the context of our nation. The present study aims to describe the epidemiology and distribution of mold infection in a tertiary care center for burn patients in Mexico City, within a period from July 2011 to July 2023.

## Methods

### Design and Clinical isolates included

This is a retrospective, cross-sectional study, from a database of the Clinical Microbiology Laboratory at Instituto Nacional de Rehabilitación Luis Guillermo Ibarra Ibarra (INR LGII) from July 2011 to July 2023, in Mexico City. Due to the absence of universally accepted diagnostic criteria for IFI in burn patients, the definition of IFI in this study was determined as the isolation of filamentous fungi from tissue culturing specimens obtained from clinically suspicious wounds. The definition proposed by the European Organization for Research and Treatment of Cancer/Mycoses Study Group: Education & Research Consortium has been developed for immunocompromised populations; however, however, its validation for burn patients remains to be determined.^14^ A similar set of diagnostic approaches has been utilized in previous studies involving burn patients. These include the IFI-BURN study^15^ and the 2013 Chinese national guideline.^16^ The study incorporated a comprehensive sample of filamentous fungi obtained from tissue biopsies of patients who had sustained burn injuries and were receiving medical care during the period of hospitalization. The identification of these molds was conducted through the application of morphology and Vitek MS (MALDI-ToF (Matrix-Assisted Laser Desorption Ionization Time-of-Flight Mass Spectrometry), Biomerieux, Marcy-l'Étoile, France). From 2011 to 2023, identification was based exclusively on morphological characteristics; however, strains were subsequently reinoculated onto Dextrose Sabouraud agar and re-identified using the Vitek MS method (MALDI-ToF, Biomerieux, Marcy-l'Étoile, France). The initial sample, which was identified as containing filamentous fungi, was retained for analysis. Subsequent samples that exhibited identical identifications were then excluded from further consideration in order to mitigate potential selection bias. The samples included in this study were tissue biopsies. Consequently, swabs and samples from the lower respiratory tract (e.g., sputum or endotracheal aspirate) lacking correlation with intrahospital pneumonia were excluded, as they are defined in guidelines as samples with low microbiological value^17^ and, in most cases, the role as a pathogen is unknown. Following the selection process, the electronic medical records were meticulously reviewed and annotated in a database. The following variables were documented: sex, age, etiology of the burn, total body surface burned area (TBSA), burn depth, and Abbreviated Burn Severity Index (ABSI). The Institutional Research Committee has formally endorsed the research project (INR LGII 114/23) as well as compliance with the personal data protection law. The requirement for informed consent was not applicable in this case, as the study exclusively utilized data from electronic medical records and did not involve any interventions with patients. Given the absence of a consensus definition for IFI in burn patients, this study defined it as any tissue biopsy with filamentous fungi isolation. Patients with incomplete electronic medical records and those colonized with molds, defined as any isolation of fungi from a superficial burn wound taken by swab and the absence of clinical manifestations, were excluded from the study.

### Statistical analysis

Continuous variables were summarized using measures of central tendency, such as the mean and median, along with measures of dispersion, including the standard deviation and interquartile range, to describe their variability. For categorical variables, frequencies and proportions were calculated to reflect their distribution within the study population. All analyses were performed using Stata v13.0 (StataCorp, TX, USA), ensuring methodological rigor and reliability in the statistical processing of the data.

## Results

### Samples included

**Figure 1.**
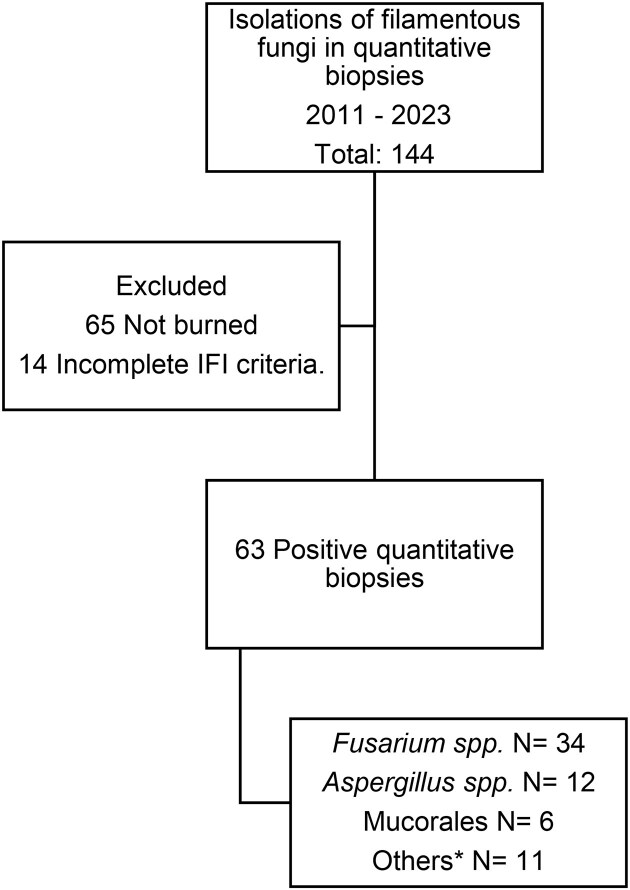
Flow chart of the study. IFI: Invasive fungal infection. *Others: *Curvularia* spp., *Cladosporium* spp., *Scedosporium prolificans* (*Lamentospora prolificans*), *Sarocladium kiliense, Alternaria alternata, Humicola fuscoatra*, and *Sordaria* spp.

A total of 144 patients with positive isolation of filamentous fungi in clinical samples were identified. A total of 65 records were excluded from the analysis due to the absence of a burn admission diagnosis. In 14 cases, the isolation of filamentous fungi was deemed to be a colonization (Fig. 1).

The study's sample population comprised 63 patients. The demographic characteristics of the study population are delineated in Table 1. The population under study was predominantly male (69.8%), and the median age was 35 years. Furthermore, 15.87% of the patients exhibited comorbidity, including three cases of diabetes (5%) and three cases of epilepsy (5%). The most prevalent etiology of burn injuries was identified as flame in 49 patients (77.78%), followed by electrical burns in 11 (17.46%). Inhalation injury was documented in 52.38% of cases. The mean percentage of total skin area affected was 46.20%, with 74.19% of burns classified as third-degree. The survival probability, as defined by the ABSI, ranged from 50% to 70%. In this sense, it is noteworthy that a total of 22 patients (35.48%) died within 60 days.

**Table 1.** Demographic and baseline characteristics.

	All patients
Variable	*N* = 63
**Age, median (±SD)**	35.08 (±18.98)
**Female *n* (%)**	19 (30.16)
**BMI median (IQR)**	27.5 (23.9–32.9)
**Comorbidities**	
Epilepsy ***n* (%)**	3 (5)
Diabetes ***n* (%)**	3 (5)
Asthma ***n* (%)**	1 (2)
No comorbidities ***n* (%)**	52 (84)
**Burn etiology**	
- Fire *n* (%)	49 (77.78)
- Scald *n* (%)	3 (3)
- Chemical *n* (%)	0 (0)
- Electrical *n* (%)	11 (17.46)
**Inhalation injury *n* (%)**	33 (52.38)
**TBSA (% Total Body Surface Area) median (range %)**	46.2 (2–95)
**Burn depth (degree)**	
- 2nd degree *n* (%)	12 (19.35)
- 3rd degree *n* (%)	46 (74.19)
- 4th degree *n* (%)	4 (6.45)
**ABSI^&^ score (survival probability)**	
- ≥99 (2–3 points) *n* (%)	2 (3.17)
- 98 (4–5 points) n (%)	6 (9.52)
- 80–90 (6–7 points) *n* (%)	18 (28.57)
- 50–70 (8–9 points) *n* (%)	17 (26.98)
- 20–40 (10–11 points) *n* (%)	13 (20.63)
- ≤10 (>12 points) *n* (%)	7 (11.11)
**Days from burn to fungal identification median (IQR)**	13 (7–19)
**Length of hospital stay, days median (IQR)**	35 (17–66)
**Mortality *n* (%)**	22 (35.48)

IQR: Interquartile range, SD: Standard deviation, BMI: Body Mass Index. ^&^ABSI: Abbreviated Burn Severity Index.

The most prevalent filamentous fungi identified were the genus *Fusarium*, detected in 34 cases (53.97%) (Table 2). Within this genus, 23 were identified as *Fusarium solani* complex (36.51%), followed by *Fusarium oxysporum* complex with 6.35% (*n* = 4). The second most prevalent genus was *Aspergillus*, identified in 12 cases (19.04%), with *Aspergillus flavus* being the most prevalent species, accounting for 9.52% of cases. Due that some strains were identified morphologically, the median interval between the onset of growth and its subsequent identification was 13 days (data not shown); time was reduced with MALDI-ToF re-identification.

**Table 2.** Isolated molds in biopsies from burned patients.

	Positive culture biopsies
Filamentous fungi	*N* = 63 (%)
*Fusarium*	
*Fusarium solani* complex	23 (36.51)
*Fusarium oxysporum* complex	4 (6.35)
*Fusarium proliferatum*	2 (3.17)
*Fusarium dimerum*	2 (3.17)
*Fusarium fujikuroi* complex	1 (1.59)
*Fusarium chlamydosporum*	1 (1.59)
*Fusarium verticillioides*	1 (1.59)
*Aspergillus*	
*Aspergillus flavus*	6 (9.52)
*Aspergillus fumigatus*	3 (4.76)
*Aspergillus niger complex*	3 (4.76)
*Mucorales*	
*Mucor circinelloides*	1 (1.59)
*Mucor spp*. (species not identified)	4 (6.35)
*Rhizopus* spp.	1 (1.59)
Other	
*Alternaria alternata*	4 (6.35)
*Sarocladium kiliense*	2 (3.17)
*Humicola fuscoatra*	1 (1.59)
*Lomentospora prolificans*	1 (1.59)
*Curvularia* spp.	1 (1.59)
*Cladosporium* spp.	1 (1.59)
*Sordaria* spp.	1 (1.59)

## Discussion

Infections in burned patients are caused in 70% of cases by bacteria and in 20%–25% of cases by fungi.^5,9^ The incidence of IFI in burn patients ranges from 6.3% to 44% in various specialized centers around the world.^7^ The most frequently described fungi include species of *Candida*, as well as the filamentous genera *Aspergillus* spp., Mucorales, *Fusarium* spp., and *Scedosporium* spp. (*Lomentospora* spp.).^8,18^ A multitude of risk factors have been identified for fungal infections in burn patients. These include advanced age, TBSA of more than 30%, deep burns, inhalation injury, a prolonged hospital stay, late wound excision, the presence of central venous catheter devices, the use of broad-spectrum antibiotics, steroid therapy, invasive mechanical ventilation, wound colonization, and immunosuppressed states.^9^ It is noteworthy that, in this study, the predominant TBSA was 46.20%, with most of them being third-degree. In a separate study, these infections were documented in patients with TBSA > 60%.^5^

Most IFI cases have been associated with thermal burns^5,7,15,19,20^; however, it is noteworthy that these pathogens may also play an infectious role in other mechanisms of injury. In a study of 749 burned patients in Mexico, 111 cases of electric burn injuries were identified, and 78% of these patients had at least one positive culture. Of these, 37/749 (4.9%) were found to contain a fungus, and 7/37 (18.9%) were identified as filamentous. In this group, three isolates of tissue biopsy were from *Alternaria* spp., two from *Fusarium* spp., and two from *Mucor* spp.^21^

The diagnosis of the infection process is challenging. A recent study proposed filamentous fungal IFI as a proven/suspected case with ≥2 tissue or bronchoalveolar lavage (BAL) cultures, or ≥2 positive blood cultures with diagnosis by Real-Time PCR (qPCR), or ≥1 positive tissue or BAL culture and ≥1 blood culture by qPCR for the same genus of filamentous fungi.^15^ In the aforementioned study, 57 of the 87 patients were diagnosed with proven/suspected IFI, and the most common agent identified was *Aspergillus* spp. (*n* = 42), followed by Mucorales (*n* = 26) and *Fusarium* spp. (*n* = 23).^15^ In Argentina, a group of 168 patients was examined, and 29 of them (17%) presented positive cultures for filamentous fungi from wound biopsies (*n* = 36) and bedsores (*n* = 2). The genera identified included *Aspergillus* spp. (24%), *Fusarium* spp. (14%), Mucorales (3%), and dematiaceous fungi (58%).^22^ Some methods have been thought of and proposed for decision making, e.g., that performed by Sarah, in which ≥2 or more blood qPCR or ≥1 culture and ≥1 qPCR to consider as predictor of IFI.^15^ Other methods suggested have been other methods used have been, galactomannan for *Aspergillus* spp.,^23^ (1→3)-β-d-glucan for *Aspergillus* spp., *Candida* spp., *Fusarium* spp., *Trichosporon* spp., *Saccharomyces cerevisiae, Acremonium* spp., *Coccidioides immitis, Histoplasma capsulatum, Sporothrix schenckii, Pneumocystis jirovecii*, however, not useful for *Cryptococcus* spp., *Blastomyces dermatitidis* (in yeast form), *Absidia* spp., *Mucor* spp., *and Rhizopus* spp..^24^

In the present study, the predominant filamentous fungal strain identified was *Fusarium* spp., accounting for nearly half of the cases (53.97%). However, temporal periods of isolation impede the formulation of hypotheses regarding outbreaks. The necessity for epidemiological molecular studies is evident in ascertaining the potential phylogenetic relationships to evaluate nosocomial acquisition. A systematic review of case reports of fusariosis in burn patients revealed 87 cases of IFI by *Fusarium* spp., with 4 cases (4/87, 4%) being reported from Mexico.^25^ This fungus is ubiquitous in the environment and distributed in water, soil, and air.^5^ In addition to the loss of the skin barrier secondary to the burn, burns in enclosed areas have recently been considered a risk factor for these infections [HR (Hazard Ratio) 1.9 (CI (Confidence Interval) 1.2–3.1)]. This is related to the concentration of viable spores found in these spaces that can be aerosolized during accidents.^15^ An additional report from Mexico indicated that hematological malignancies and burned patients are two primary risk factors associated with fusariosis.^26^ The objective of this study is to contribute to the microbiological epidemiology of IFI in patients with burns. This area of research has been understudied in our country due to the limited number of reference centers and the challenges in adequately culturing and identifying these agents. In comparison with other series,^15,19^ the sample size is considerable, particularly in light of the challenges and unavailability of these molds in numerous medical centers.^7^

It is imperative to acknowledge that this information is derived from a single center; however, it is a reference hospital, and therefore, some patients may have been referred from other hospitals.

Therefore, this is a single-center cohort designed to appraise the epidemiology of IFIs in patients with deep burn wounds. The epidemiological findings differ from the rest of the world, where *Fusarium* spp. is the most prevalent mold species isolated. In the treatment of such patients, it is imperative to suspect the presence of this particular fungus in order to initiate antifungal treatment and wound debridement promptly. The mortality rate documented in this report was 22%, underscoring the critical importance of establishing a comprehensive protocol for the evaluation of burn patients, the implementation of an early surgical approach, the use of early molecular methods and markers, and the administration of timely antifungal treatment. Conversely, this percentage could also be indicative of an underestimation associated with the limitations of the methods, the absence of pathology information, and the lack of early markers in our field.
